# Ethylene Sensor-Enabled Dynamic Monitoring and Multi-Strategies Control for Quality Management of Fruit Cold Chain Logistics

**DOI:** 10.3390/s20205830

**Published:** 2020-10-15

**Authors:** Xuepei Wang, Xinwu Li, Daqi Fu, Rajko Vidrih, Xiaoshuan Zhang

**Affiliations:** 1 Beijing Laboratory of Food Quality and Safety, College of Engineering, China Agricultural University, Beijing 100083, China; SY20193071259@cau.edu.cn (X.W.); XinwuLi@cau.edu.cn (X.L.); 2 College of Food Science & Nutritional Engineering, China Agricultural University, Beijing 100083, China; daqifu@cau.edu.cn; 3 Department of Food Science and Technology, Biotechnical Faculty, University of Ljubljana, Ljubljana 999151, Slovenia; Rajko.Vidrih@bf.uni-lj.si

**Keywords:** ethylene sensor, dynamic monitoring, multi-strategies control, postharvest quality, fruits, cold chain

## Abstract

Due to the presence of bioactive compounds, fruits are an essential part of people’s healthy diet. However, endogenous ethylene produced by climacteric fruits and exogenous ethylene in the microenvironment could play a pivotal role in the physiological and metabolic activities, leading to quality losses during storage or shelf life. Moreover, due to the variety of fruits and complex scenarios, different ethylene control strategies need to be adapted to improve the marketability of fruits and maintain their high quality. Therefore, this study proposed an ethylene dynamic monitoring based on multi-strategies control to reduce the post-harvest quality loss of fruits, which was evaluated here for blueberries, sweet cherries, and apples. The results showed that the ethylene dynamic monitoring had rapid static/dynamic response speed (2 ppm/s) and accurately monitoring of ethylene content (99% accuracy). In addition, the quality parameters evolution (firmness, soluble solids contents, weight loss rate, and chromatic aberration) showed that the ethylene multi-strategies control could effectively reduce the quality loss of fruits studied, which showed great potential in improving the quality management of fruits in the supply chain.

## 1. Introduction

Ethylene (C_2_H_4_) is a phytohormone which has numerous effects on fruit growth, development, post-harvest quality and storage life [1,2]. The ethylene synthesis peak appears during the ripening of climacteric fruits, which promotes the ripening of fruits. Hence, the gaseous ethylene emissions reflect the maturity stage of fruits and drive dramatic changes during fruit ripening [3], such as promoting fruit color changes, softening, and increasing fruit aroma and affecting texture [4]. Additionally, ethylene also helps the conversion of starch to sugar, resulting in increasing the sugar content of fruits. However, the negative effects of ethylene on quality also focus on accelerating the natural processes of fruit development, ripening, and senescence. Even though non-climacteric fruits, such as sweet cherries [5], grapes [6], and citrus [7], do not produce autocatalytic ethylene, exogenous ethylene also has an important effect on their quality and storage life. Thence, it is of paramount importance to monitor and control the ethylene mission rate in the fruit supply chain to optimize fruit freshness, which has attracted lots of attention around the world [8,9]. The cold chain logistics is a vivid example, which is aimed to promote delay in ethylene production (DEP) and slow down the metabolism (such as respiration and hydrolysis) by precise temperature control [10,11]. In addition to the above active temperature control, many scholars are also devoted to passive protection measures to reduce quality loss, such as modified atmosphere preservation, radiation preservation, microbial preservation, application of coating, and chemical sterilization [12,13,14]. In some scenarios of certain fruits (such as bananas, mango, and kiwi fruits), the ethylene content (including endogenous ethylene and exogenous ethylene) needs to be increased to accelerate fruit ripening, thereby meeting flexible market demand.

Thought the above analysis, the ethylene content in the post-harvest fruit microenvironment needs to be accurately monitored firstly. The data collection technologies used in fruit cold chain logistics have evolved from the traditional barcodes or recorder technology to the modern IoT monitoring system with wireless communication technology and sensor technology, which all have the characteristics of intelligence, miniaturization, and real-time analyze. Owing to the easy deployment and applicability, the wireless sensor network (WSN) technology, as a typical representative technology, holds a leading position in precision agriculture monitoring and environment monitoring [15,16]. More specifically, the monitoring of ethylene gas in the fruit microenvironment mainly relies on gas chromatography procedure, electrochemical sensing, and optical sensing. Among them, electrochemical sensing has good repeatability, accuracy, and fast response/recovery characteristics, becoming the mainstream technology for ethylene monitoring [17].

In addition to the abovementioned ethylene monitoring, accurate and effective ethylene control methods cannot be ignored for both endogenous and exogenous ethylene. Endogenous ethylene is synthesized internally by the fruit itself, released into the microenvironment and will accelerate fruit ripening and aging, while exogenous ethylene is mainly added manually. Therefore, to retard fruit ripening and senescence, it is necessary to inhibit the synthesis of endogenous ethylene and reduce the level of exogenous ethylene in the fruit microenvironment. On the other hand, in order to ripen the fruit, adding exogenous ethylene and promote the biosynthesis of endogenous could be adopted.

Although some scholars have conducted in-depth researches on ethylene, those studies lack the variety of fruit varieties and corresponding control strategies, especially fail to integrate with ethylene monitoring. This research contributes to the field of study in the following ways. First, an ethylene dynamic monitoring system was built based on electrochemical ethylene sensors. Second, the ethylene sensing characteristics and monitoring performance were analyzed. Finally, the multi-scenario ethylene control strategy has been tested and verified in various fruits and scenarios, proving its effectiveness in post-harvest fruit quality management.

## 2. Materials and Methods

### 2.1. Conceptual Framework

**Figure 1:**
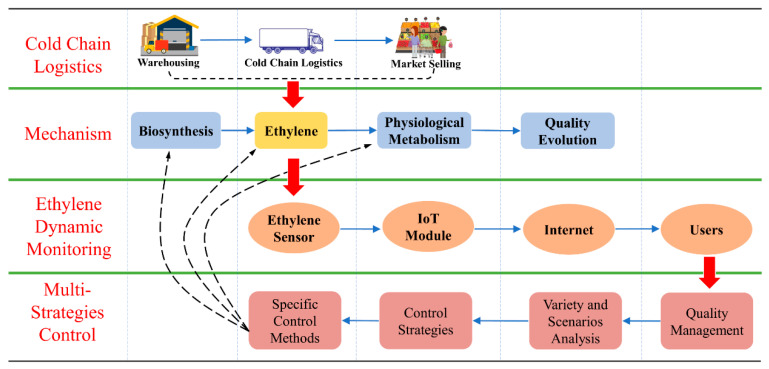
Conceptual framework of ethylene dynamic monitoring enabled multi-strategies control.

The ethylene sensor enabled dynamic monitoring and multi-strategies control for quality management of the fruit cold chain mainly consists of four parts. Figure 1 shows its conceptual framework.

#### 2.1.1. Cold Chain Logistics

Fruits are generally placed in the cold storage for pre-cooling after harvesting, then transported to different sales markets by cold chain trucks according to different market needs. Ethylene plays a critical role during the logistics process, which has an important impact on the quality of fruits [4].

#### 2.1.2. Ethylene Mechanism

Through a series of biosynthetic pathways, ethylene is generated inside the fruit and gradually released into the microenvironment, which has a critical impact on the post-harvest fruit’s physiological metabolism and ultimately affects the fruit’s aging and quality evolution [18,19].

#### 2.1.3. Dynamic Monitoring

Since ethylene has an important effect on fruit ripening and quality evolution in fruit cold chain logistics, it is necessary to monitor it accurately and in real-time. Ethylene is monitored by electrochemical sensors, and ethylene data is transmitted to the Internet terminal in real-time through the Internet of Things (IoT) module, which could provide users with decision-making supports.

#### 2.1.4. Multi-Strategies Control

Based on the above ethylene monitoring data, users could flexibly adapt different ethylene control strategies. According to different fruit varieties and scenarios characteristics, the appropriate control strategy could be determined, then choosing specific ethylene control methods to achieve flexible maturity control and quality management of fruit.

### 2.2. Mechanism Analysis

**Figure 2:**
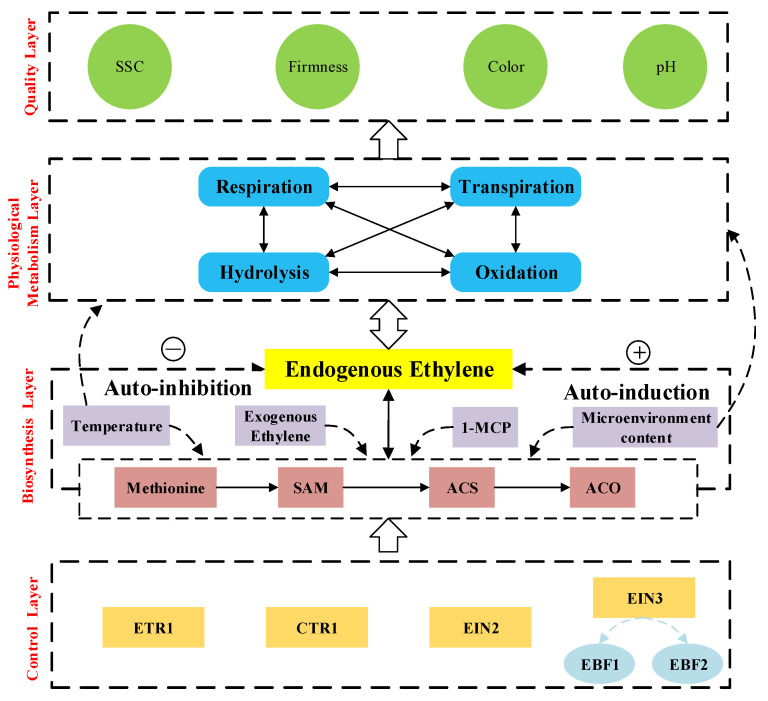
Biosynthetic pathway of ethylene and effects on fruit quality based on [11,19,20,21]. *ETR1*: Ethylene Resistance 1; *CTR1*: Constitutive Triple Response 1; *EIN2*: Ethylene Insensitive 2; *EIN3*: Ethylene Insensitive 3; *EBF1*:EIN3-binding F box protein 1; *EBF2*: EIN3-binding F box protein 2; *SAM*: S-adenosylmethionine; *ACS*: 1-aminocyclopropane-1-carboxylic acid synthase; *1-MCP*: 1-Methylcyclopropene; *ACO*: 1-aminocyclopropane-1-carboxylic acid oxidase.

The purpose of controlling microenvironment ethylene content is to allow longer storage periods and provide the best quality for consumers over the entire year. Hence, the ethylene biosynthesis pathway within the fruit and how it subsequently affects the fruit quality should be enlightened first. The biosynthetic pathway of ethylene and its effect on fruits quality proposed in Figure 2 is based on references [11,19,20,21] and mainly includes four layers:

➣ *Control Layer*: Fruit genes control all life activities and trait expression of fruits. Some genes proteins related to ethylene synthesis, including ETR1, CTR1, EIN2, and EIN3 [21]. These proteins could respond to different ethylene levels inside the fruit, controlling the ethylene biosynthesis.
➣ *Biosynthesis Layer*: Under the stimulation of above control proteins, the methionine is converted into S-adenosylmethionine, which is subsequently converted to ethylene by means of enzymes ACS, and ACO [11,19,20]. The biosynthesized ethylene itself also strongly regulates the expression and activity of ACS and ACO, thereby achieving the feedback regulation [18,21].
➣ *Physiological Metabolism Layer*: Endogenous ethylene regulates a series of physiological and metabolic activities of fruits, including respiration, transpiration, hydrolysis, and oxidation [22]. For example, for climacteric fruits, the ethylene content rises rapidly during the maturity stage, the respiration rate of the fruit also increases almost exponentially.
➣ *Quality Layer*: These physiological and metabolic activities of fruits directly affect the fruit quality, including firmness, SSC, pH, chromatic aberration, etc [23]. For example, the respiration of the fruit consumes the starch and converts it to fructose, thus resulting in a decrease of firmness and the increase of pH.

### 2.3. Ethylene Dynamic Monitoring

#### 2.3.1. Design of Ethylene Dynamic Monitoring

**Figure 3:**
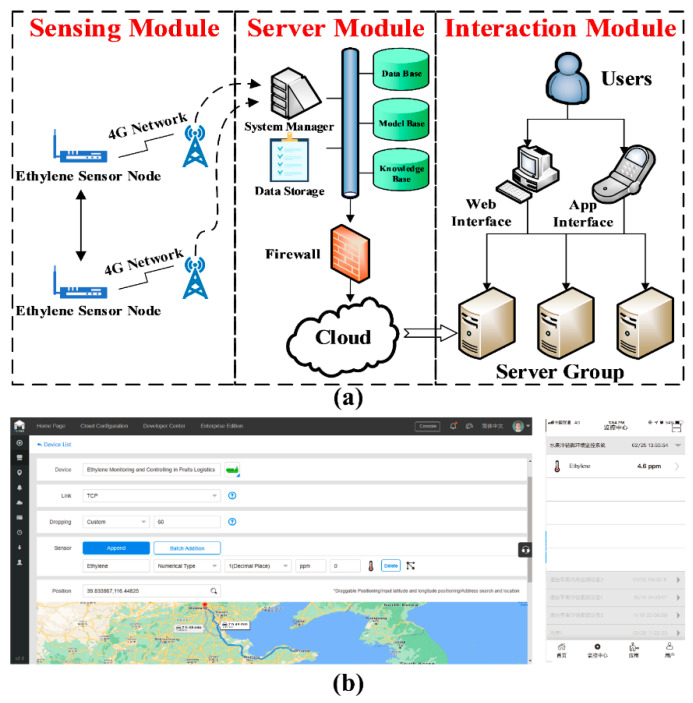
Ethylene dynamic monitoring. (**a**) The framework of the ethylene dynamic monitoring; (**b**) Web and App interface.

The ethylene dynamic monitoring mainly includes three modules (as shown in Figure 3a):

The *Sensing Module* is mainly responsible for collecting microenvironment ethylene data, which mainly includes the ethylene sensor, micro-control chip, and IoT unit. The micro-control chip controls the sensor node for data collection (such as collection time and frequency) and transmits the ethylene data to the management server through IoT 4G communication.

The *Server Module* is the channel connecting the ethylene sensor node and the users’ interaction. It is responsible for data management sent by the ethylene sensor, such as data base, model base, and knowledge base, which could provide a reference or decision-making for stakeholders.

The *Interaction Module* is mainly responsible for providing the user with interaction, including Web interface and App interface (shown as Figure 3b), which could provide staff with functions such as equipment management, editing, data query, chart display, and warning notification.

#### 2.3.2. Implementation of Ethylene Dynamic Monitoring

**Figure 4:**
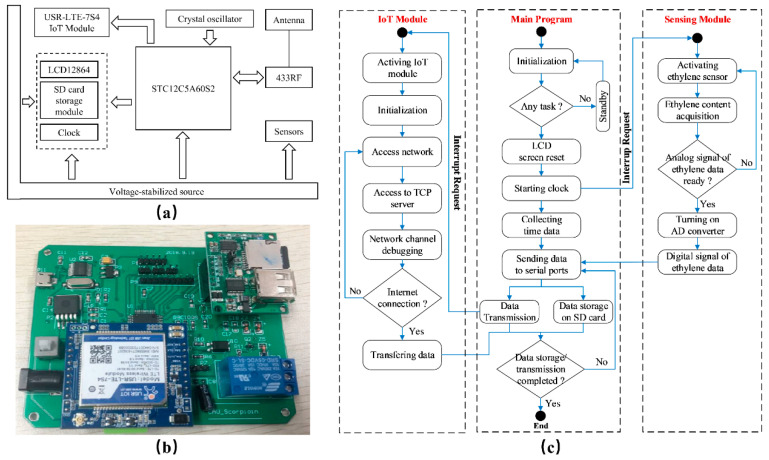
Design and implementation of the ethylene sensor node. (**a**) The framework of hardware design; (**b**) Physical implementation; (**c**) Software implementation.

The ethylene sensor node integrated the main control unit, power supply unit, sensor acquisition unit (produced by Beijing AnJieDa Technology Co., Ltd., Beijing, China), transmission unit, clock unit, relay control unit, storage unit, and display unit. From the perspective of the miniaturization, easy implementation, low power consumption, low cost, the STC12C5A60S2 main control chip developed by Hongjing Technology (Jinan, China) was used and the transmission module applied the USR-LTE-7S4 4G wireless transmission module (produced by YOUREN Company, city, country), which realizes 4G network communication. The hardware of the ethylene sensor node as shown in Figure 4a,b. The software implementation of the ethylene monitoring relies on KEIL UVISON4 (Keil Software, ARM Limited, Germany) the development language was C language. The system software process flowchart is shown in Figure 4c.

### 2.4. Design and Methods of Experiment

#### 2.4.1. Static Calibration Experiment of Ethylene Sensor

**Figure 5:**
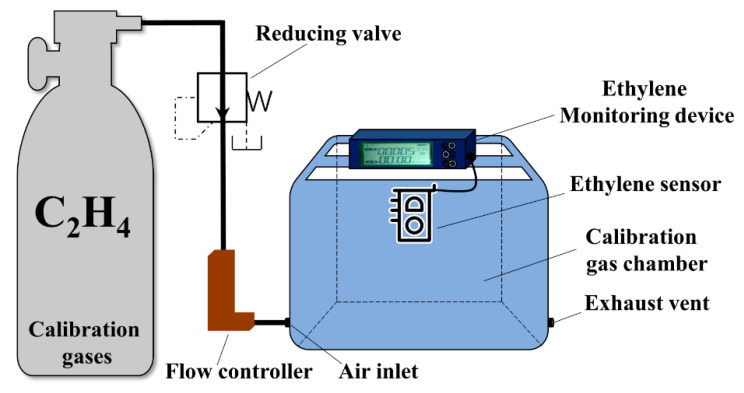
Experimental setup of the ethylene sensor calibration test.

The calibration platform for the ethylene sensor is shown in Figure 5. Different concentrations of standard ethylene from gas cylinders (mixtures of ethylene and nitrogen) were used for calibration. Firstly, opening the air chamber for 15 min to fully expose the ethylene sensor to the air environment. Then, sealing the gas chamber and opening the calibration ethylene gas tank. By adjusting the pressure-reducing valve and the flow controller, the different concentrations of ethylene gas could enter the sealing gas chamber at a flow rate of 500 mL/min for 15 min. Finally, the stable voltage value was recorded, and opening the air chamber sealing cover to fully expose the gas sensor to the air until the sensor signal returns to the basic value.

**Figure 6:**
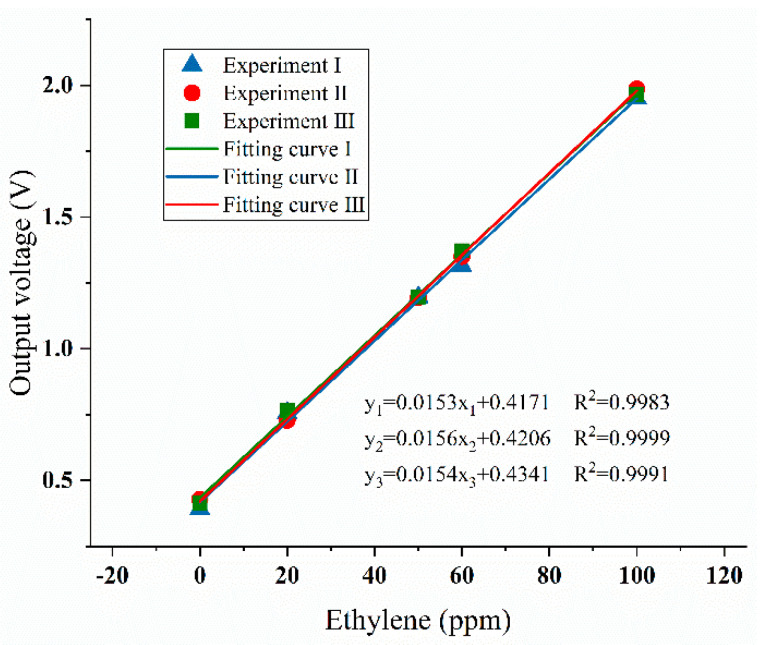
The calibration curves of electrochemical ethylene sensor.

The ethylene sensor was calibrated in the sealed gas chamber at different concentrations of ethylene gas (0, 20, 50, 60, and 100 ppm), and the corresponding response voltage of the ethylene sensor were recorded, the electrochemical ethylene sensor was repeatedly tested three times, and the calibration results are shown in Figure 6. The three fitting curves all showed that the ethylene gas sensor had good linearity (R^2^ > 0.99) and repeatability.

#### 2.4.2. Dynamic Response Experiment of Ethylene Sensor

In order to comprehensively explore the performance and characterization of the ethylene sensor, the dynamic response experiment was conducted on the ethylene sensor with 20 ppm standard ethylene gas. By integrating and differentially transforming the dynamic response time-domain curve of the ethylene sensor, the key time-domain characteristic parameters of the ethylene sensor could be extracted, including amplitude accumulation of sensing signal, response speed, recovery speed, response acceleration, recovery acceleration, etc., These parameters could comprehensively and accurately evaluate the performance ethylene sensor. The specific calculation formulas are shown in Equations (1)–(3):

Integral formula:

(1) $$I_{t}=\int_{0}^{t}C_{t}$$

First-order differential formula:

(2) $${D^{\prime }}_{t}=\frac{\partial \sigma_{t}}{\partial t}$$

Second-order differential formula:

(3) $${D^{\prime \prime }}_{t}=\frac{\partial^{2}\sigma_{t}}{\partial t^{2}}$$

where, $C_{t}$—the voltage signal of the ethylene sensor at time t; $\partial_{t}$—the voltage signal of the ethylene sensor at time t after filtering.

#### 2.4.3. Verification Experiment

The blueberries experiment aimed to simulate the cold chain logistics of climacteric fruits. A total of about 3600 g of blueberries were divided into part A (for ethylene monitoring) and part B parts (for quality parameter determination). Part A was further divided into three groups, from A1 to A3 (600 g each group), and they were all placed in 18. 2 × 18. 2 × 10. 8 cm containers sealed with a PE film (0.02 mm). These blueberry samples were placed in a thermostat at 0, 5, 22 °C respectively (with 90% humidity), and the ethylene acquisition data frequency was 10 min. Part B was also divided into three groups, then each group is equally divided into six packages (100 g each), which were placed in a 1/6 size sealed box of the above-sealed containers sealed with PE film (0.02 mm). A total of 45 blueberries were randomly selected as samples for daily quality parameter determination. Each quality parameter measurement was conducted three times, and the average value was taken as the final value.

The sweet cherries experiment aimed to simulate the cold chain logistics of non-climacteric fruits. A total of 66 kg of sweet cherries of similar size and maturity (90% of commercial maturity) were selected and divided into three groups: I, II, and III. For each group, sweet cherries were divided into 11 separate sections, of which 10 were used for quality parameters determination and the 11th for ethylene gas monitoring. Group I was used to simulating ambient temperature transportation. Group II was used to simulate the ice-added transportation (two 200 mL ice packs were placed in each separate package). Group III was used to simulate precooling transportation and was placed in a refrigerated warehouse (precooling at 0–1 °C for 12 h). The acquisition frequency of the ethylene sensor was 27 s, and these quality parameters were determined twice a day at 8 am and 5 pm, respectively.

In the apple experiments: (1) 250 Fuji apples (picked from Yantai City) were randomly divided into two groups (experimental group and control group). The apples in the experimental group were sealed and fumigated with 1 μL/L 1-MCP for 12 h, while the control group was only sealed for 12 h. 1-MCP is an ethylene inhibitor which binds to ethylene receptors inhibiting ethylene action. Apples were stored at room temperature. In each experiment, twenty apples were randomly selected from each group and put into a sealed container 2 h for microenvironment ethylene content monitoring. Every four days, fifteen fruits were randomly selected from the two groups for quality parameter determination. (2) 220 Fuji apples were randomly divided into two groups (experimental group and control group). The Fuji apple of the experimental group was sealed with 20% carbon dioxide, while the control group was sealed with air. Among them, twenty apples were randomly selected from each group and packaged with 20% carbon dioxide or air for continuous ethylene content monitoring. Every three days, fifteen fruits were randomly selected from the two groups for quality parameter determination.

#### 2.4.4. Quality Parameters Determination Methods

The methods and devices used for quality parameters determination were as follows:

(1) Firmness. The firmness was measured by the FHT-05 Firmness Tester (produced by Landtek Co., Ltd., Guangzhou, China). Fifteen experimental samples were randomly selected for measurement, and two symmetrical parts were taken for each fruit and then averaged.
(2) Soluble solids contents (SSC). Fifteen experimental samples were randomly selected, the juice was squeezed with gauze, and the soluble solids content was determined using an ATAGO PAL-1 digital refractometer (manufactured by ATAGO Company, Guangzhou, China).
(3) pH. The pH was determined with a pH digital acid meter (produced by Testo SE & Co. KgaA, Shanghai, China). Fifteen experimental samples were randomly selected, and the average value was obtained after two determinations.
(4) Weight loss rate. The weight loss rate was determined by the weighing method [24], the calculation formula was as follows:
  (4) $$R_{w}=\frac{W_{o}-W_{f}}{W_{o}}\times 100\%$$
  where, $R_{w}$—weight loss rate; $W_{o}$—origin weight; $W_{f}$—final weight.
(5) Rotting rate. Rotten fruit means that there is at least one lesion on the surface of the fruit or leakage of juice, softening, shrinkage, or rot of the fruit [25]. The calculation formula was as follow:
  (5) $$\varepsilon =\frac{m_{1}}{m_{2}}\times 100\%$$
  where, $m_{1}$—the weight of rotten fruit; $m_{2}$—total weight of fruits; ε-the rotting rate.
(6) Chromatic aberration. The chromatic aberration was measured by a CR-410 chromatic aberration analyzer (produced by Konica Minolta Co., Ltd., Japan, Tokyo). The final value of the L*, a*, and b* were obtained by averaged three measurement results. The total chromatic aberration ΔE was calculated by the following formula [26]:
  (6) $$\Delta E=\sqrt{{\left(L*\right)}^{2}+{\left(a*\right)}^{2}+{\left(b*\right)}^{2}}$$

## 3. Results and Discussion

### 3.1. Characteristic Analysis of Ethylene Dynamic Monitoring

**Figure 7:**
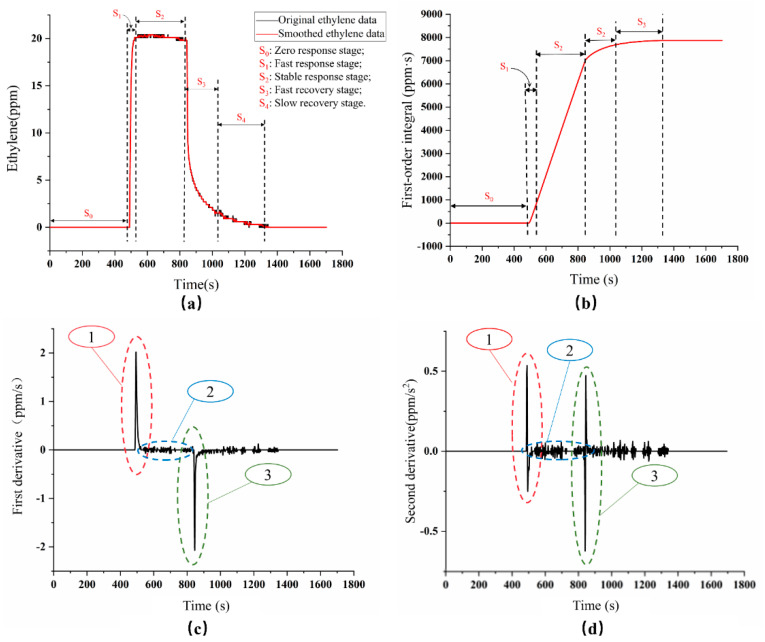
Characteristic analysis of the ethylene dynamic monitoring: (**a**) ethylene dynamic response curve; (**b**) first-order integral curve; (**c**) first-order derivative curve; (**d**) second-order derivative curve.

Different time-domain characteristic parameters could be extracted from the dynamic response curve of the ethylene sensor, which can fully reflect the performance of the ethylene sensor and the microenvironment ethylene content variation information [27,28,29]. Therefore, the complete response curve of ethylene dynamic monitoring in a 20 ppm ethylene environment (the calibrated gas was a mixture of ethylene and nitrogen) was explored. The ethylene response curve was shown in Figure 7a, which could be divided into five stages from S_0_ to S_4_.

➣ S_0_ stage: The S_0_ stage was the zero-response stage of the ethylene sensor in the air, also known as the baseline value. From the response curve, it could be seen that the response value of the ethylene dynamic monitoring was 0 ppm in the air, which was consistent with the actual ethylene concentration in the atmosphere.
➣ S_1_ stage: The S_1_ stage was the fast response stage. When the 20 ppm of standard calibration ethylene gas (mixture of with ethylene and nitrogen) was added into the gas chamber at a rate of 500 mL/min through the flow controller, the ethylene gas sensor responded quickly to ethylene gas and reached 90% of the maximum response value after 18 s, which was called response time.
➣ S_2_ stage: The S_2_ stage was called the stable response stage, namely the ethylene concentration in the gas chamber had reached a stable state. From the time-domain curve, it could be seen that the ethylene dynamic monitoring reached the maximum response value (20.40 ppm) at about 600 s, and then kept the dynamic stability at 20.13 ppm. Therefore, it could be considered that the ethylene sensor showed a good dynamic response.
➣ S_3_ stage: The S_3_ stage was the fast recovery stage. In this stage, the air chamber cover of the experimental device was opened to make the ethylene sensor fully expose to the air. From the time-domain curve, it could be seen that the value of the ethylene sensor decreased rapidly to 30% of the maximum response value during the 58 s.
➣ S_4_ stage: The S_4_ phase was a slow recovery stage. At this time, the value of the ethylene dynamic monitoring dropped slowly. It took about 100 s to fully recover to the baseline value. The possible reason may be that there was still a small amount of ethylene gas around the sensor, which had a limited impact on the ethylene sensor (influence of residual gas on maximum ethylene sensor response was less than 1%).

Since the cumulative change in response amplitude, response change speed, response recovery speed, and acceleration of the sensor cannot be obtained from the time-domain characteristic curve, the first-order integral, first-order differentiation, and second-order differentiation were performed on the time-domain curve to obtain the first-order integral curve, first-order derivative curve, and second-order derivative curve, as shown in Figure 7b–d. A series of characteristic parameters were summarized in Table 1. 

It can be seen from the integral curve that the maximum signal cumulation of the ethylene sensor reaches about 7800 ppm·s during the whole test periods, and the accumulation of the sensor’s response amplitude reaches 7000 ppm·s during the response and stability stage (from gas entry to gas exit).

The response/recovery speed and response/recovery acceleration of the sensor can be obtained from the differential curve (Figure 7c,d). More specifically, the maximum response speed and recovery speed of the ethylene dynamic monitoring were 2.3986 ppm/s and −2.4043 ppm/s, respectively. According to the second-order derivative curve (Figure 7d), the maximum acceleration value of the ethylene dynamic monitoring response was 0.8586 $\mathrm{ppm}/s^{2}$, and the minimum change value of acceleration was −0.9979 $\mathrm{ppm}/s^{2}$, which indicated that the ethylene gas sensor had a high response and could closely monitor the change of ethylene concentration in the fruit microenvironment.

### 3.2. Ethylene Multi-Strategies Control: Low Temperature

#### 3.2.1. Blueberry Experiment

The ethylene evolution of blueberries is shown in Figure 7a. The ethylene content (<5 ppm) in group A (0 °C) and group B (5 °C) was far lower as compared to group C (22 °C) (>25 ppm). The possible reason was the low temperature that inhibited the ethylene biosynthesis and physiological metabolism in general of blueberries fruit effectively, thereby reducing the release of endogenous ethylene.

**Figure 8:**
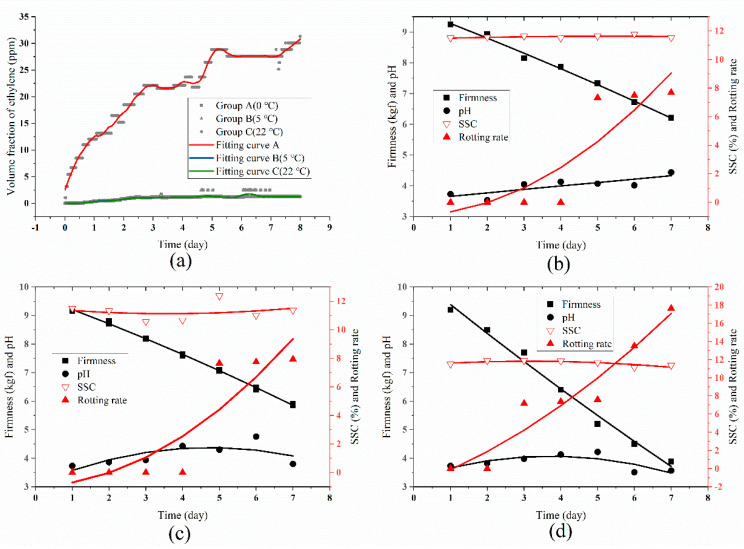
Data analysis of blueberries experiment. (**a**) The comparison curve of ethylene content of blueberries in three different experimental temperatures (the curves of group A and group B have almost coincided); (**b**–**d**) The quality parameters evolution of blueberries in group A (0 °C), group B (5 °C), and group C (22 °C).

To further validate the above analysis, the quality parameters of blueberries (firmness, SSC, pH, and rotting rate) were measured three times every day, and the average values of these data was considered as the final value, which is shown in Figure 8b–d. The general evolution trend of these quality parameters in both three groups was similar. More specifically, the blueberries in group C showed the lowest firmness (3.88 kgf) and the highest spoilage (17.65%) as compared to that of group A and group B after 7 days, while the SSC and pH showed no significant difference among the three groups. The correlation analysis (as shown in Table 2) of ethylene concentration and quality parameters of blueberries also presented the same results. The ethylene concentration and firmness showed a high negative correlation (−0.99~−0.96), the ethylene concentration and rotting rate showed a high positive correlation (0.80~0.92). However, the correlation between ethylene concentration and SSC or pH was not obvious.

#### 3.2.2. Sweet Cherries Experiment

**Figure 9:**
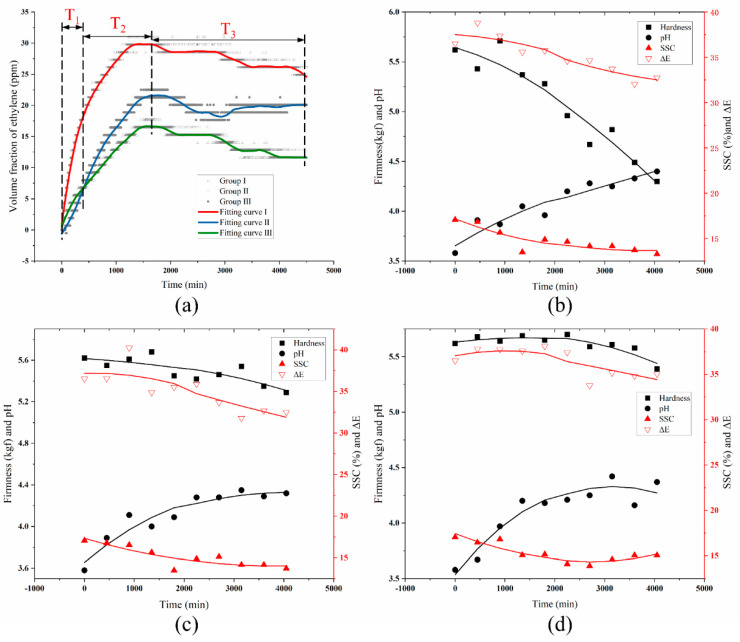
The data analysis of sweet cherries experiment. (**a**) Comparison curve of ethylene content of sweet cherries in three groups. (**b**–**d**) The quality parameters evolution of sweet cherries in group I, group II, and group III.

The changes in ethylene contents in the three groups all showed a roughly upward trend (shown in Figure 9a), but the ethylene content in Group I was higher than that of the other two groups during the entire experiment period. Specifically, the entire evolution could be divided into three time periods: T_1_, T_2,_ and T_3_. In the T_1_ Period (from 0 to 500 min), the ethylene concentration in Group III was higher than that of Group II then reached an equivalent content (6 ppm) at about 500 min. In the following T_2_ Period (from 500 to about 1700 min), the ethylene concentration continued to rise and reached a peak at 1700 min, the specific value was 30 ppm (Group I), 20 ppm (Group II), and 15 ppm (Group III) respectively. In the next T_3_ Period, the ethylene concentration was in a state of slow change in both of three groups. The results showed that the low temperature obtained with ice-addition, or precooling treatments, could effectively inhibit the production of ethylene in sweet cherries.

The quality parameters evolution (the results represent average of three measurements) of sweet cherries were shown in Figure 9b–d. The curves of firmness, SSC, and ΔE showed a downward trend, and the pH curve showed an upward trend. To specifically explore the relationship between the quality parameters and the ethylene contents, the correlation analysis was conducted (as shown in Table 3), which showed that the firmness, SSC, and ΔE correlated negatively to the ethylene concentration change, while the pH correlated positively. Comparing correlation results of the sweet cherries to the blueberries experiment, the sweet cherries correlation coefficient of firmness (or ΔE) and the ethylene lower than 0.5, while that of SSC and pH to the ethylene was statistically significant (correlation coefficient >0.5).

### 3.3. Ethylene Multi-Strategies Control: 1-MCP

**Figure 10:**
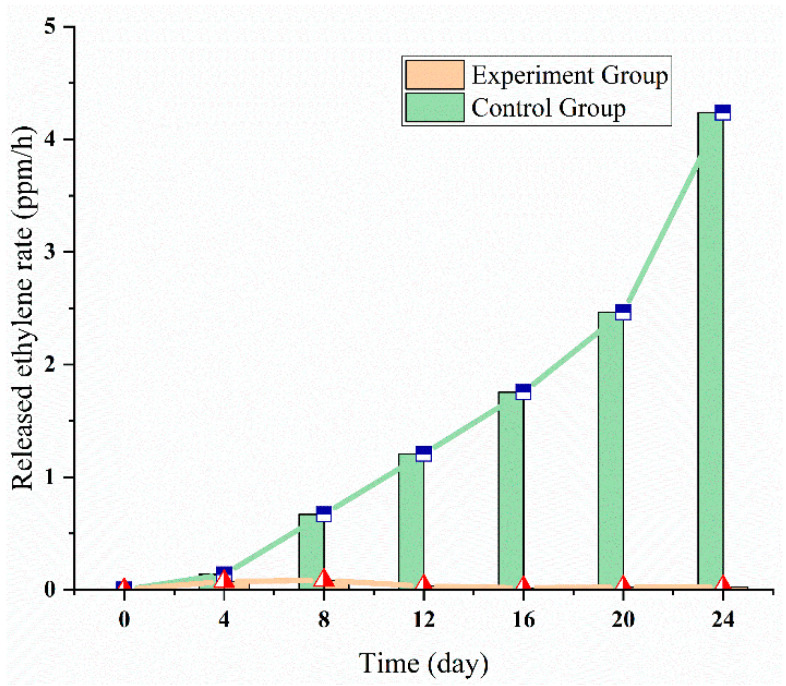
The released ethylene rate of apple experiment with 1-MCP treatment.

In the apple experiment (1), the monitored ethylene data was preprocessed to observe the effect of ethylene control strategies, the results were shown in Figure 10. The released ethylene rate in the control group showed an upward trend, while the released ethylene rate in the experimental group almost remains at 0 ppm/h level. At the end of the experiment, the released ethylene rate in the control group reached about 4 ppm/h, which is 40 times lower than that in the experimental group (<1 ppm/h). The possible reason is that 1-MCP inhibited the activity of ACS and ACC in the ethylene biosynthesis pathway. Therefore, the 1-MCP effectively inhibited the ethylene synthesis in apple fruits.

**Figure 11:**
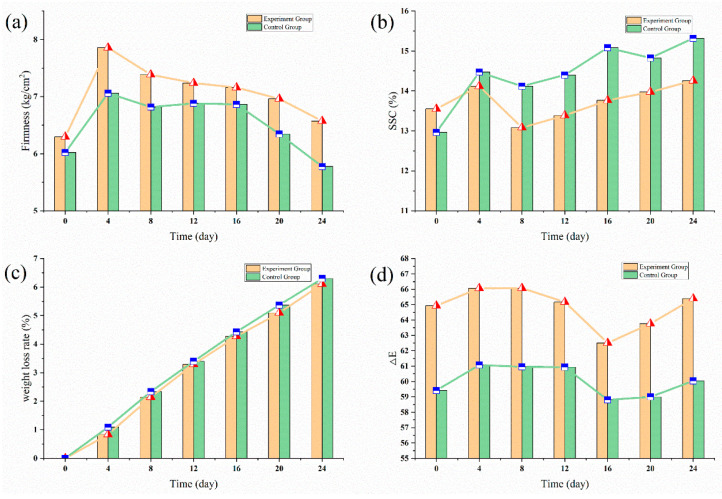
The quality parameters change of apples with 1-MCP treatment. (**a**) Firmness evolution of apples; (**b**) Soluble solids contents evolution of apples; (**c**) Weight loss rate changes of apples; (**d**) chromatic aberration (ΔE) evolution of apples.

To evaluate the performance of the ethylene multi-strategies control more intuitively, apples’ quality parameters were determined, including firmness, SSC, weight loss rate, and chromatic aberration (ΔE), the results were shown in Figure 11.

Except for the weight loss rate which was shown in Figure 11c (where there was no obvious difference), the apples of the experimental group could maintain better quality than those of the control group in terms of firmness, SSC and ΔE. Specifically, from the Figure 11a,b, apples with 1-MCP treatment maintained higher firmness (on average 8.17% higher) and lower soluble solid contents (on average 4.80% lower) throughout the experiment period. As for chromatic aberration (Figure 11d), the 1-MCP-treated apples showed a higher ΔE value (on average 8% higher), which means that the 1-MCP treatment could better maintain apples original color. Therefore, the 1-MCP treatment could effectively inhibit the endogenous ethylene synthesis, and thus keeping better quality.

### 3.4. Ethylene Multi-Strategies Control: Modified Atmosphere Package (20% CO_2_)

**Figure 12:**
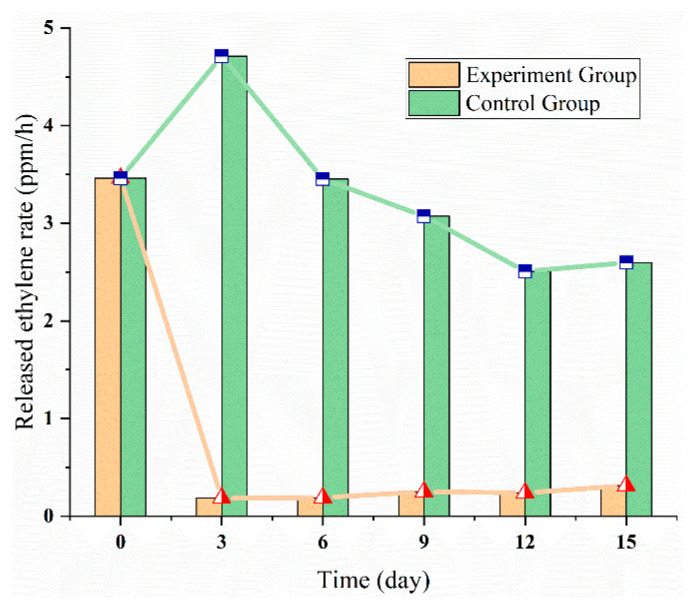
The released ethylene rate of apple experiment with 20% CO_2_.

In addition to the application of 1-MCP to inhibit apple ethylene synthesis, the effect of modified atmosphere package (MAP) with 20% CO_2_ concentration on apples’ ethylene synthesis and quality were also explored. The curve of the released ethylene rate was shown in Figure 12. Throughout the experiment period, the released ethylene rate in the experimental group was lower than that of the control group. The released ethylene rate in the control group peaked on the third day (approximately 5 ppm/h), then decreased and finally remained stable at 3 ppm/h. The possible reason for the decrease may be the oxygen in the sealed packaging was exhausted, inhibiting the general metabolism of fruits, including the biosynthesis and release of ethylene. The ethylene emission rate in the experimental group dropped sharply after putting into 20% CO_2_ MAP, and reached the lowest value (about 0.5 ppm/h) on the third day, then remained at this level. The possible reason was that high levels of CO_2_ may inhibit the conversion of ACC to ethylene. Hence, the MAP could effectively inhibit apple’s ethylene synthesis.

**Figure 13:**
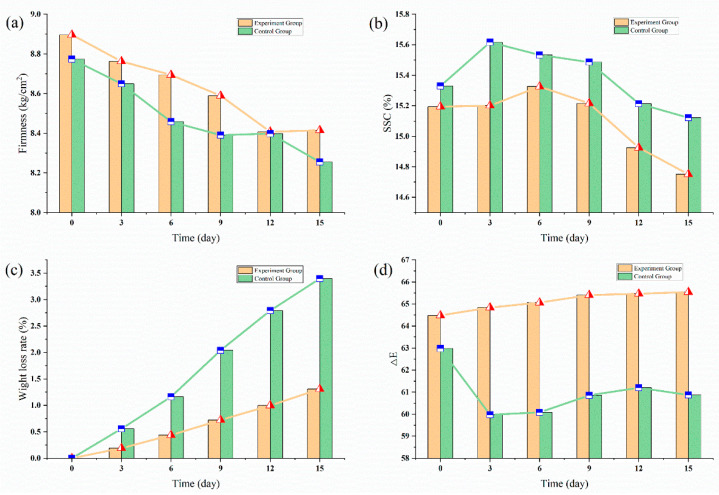
The quality parameters evolution of Fuji apples with 20% CO_2_ treatment. (**a**) Firmness evolution of apples; (**b**) Soluble solids contents evolution of apples; (**c**) Weight loss rate changes of apples; (**d**) chromatic aberration (ΔE) evolution of apples.

Similarly, the four quality parameters were also determined, the results were shown in Figure 13. The results all showed that the modified atmosphere packaging (MAP) with 20% CO_2_ could maintain better apple quality. More specifically, apples in MAP could maintain higher firmness and ΔE, on average 1.65% and 6.82% respectively higher than that of the control group, which were shown in Figure 13a,d. Besides, apples in the MAP had lower SSC and weight loss rates, which were 1.83% and 64% respectively lower than that of the control group (as shown in Figure 13b,c). Therefore, the MAP could effectively inhibit the endogenous synthesis of apples and delay the quality decline, which also reflected the effectiveness of the proposed ethylene multi-strategies control.

### 3.5. Analysis of Multi-Strategies Ethylene Control

Although the three common ethylene control methods were discussed above with typical fruit varieties, in order to meet the requirements of ethylene control in comprehensive varieties of fruits and scenarios, the multi-strategies ethylene control was supplemented and analyzed combined with relevant literature (as shown in Table 4).

#### 3.5.1. Strategy I—Adding Ethylene for Climacteric Fruits

The strategy I mainly serves for climacteric fruits that need artificial ripening, the representative fruits including banana, mango, and kiwi fruit. Owing to the requirements of long-term storage or transportation, these fruits are usually picked before fully expressed physiological maturity in a so called climacterium minimum phase. Therefore, these fruits generally need to be ripened to achieve optimum ripening and eating quality. The most common control strategy is adding microenvironment ethylene content. Hence, the most direct control method is controlling relay to automatically release exogenous ethylene gas, which could be applied in fruit storage integrated with automatic control technology.

Another popular control method is the application of ethephon, which is a plant growth stimulant that not only breaks down into ethylene (pH > 4), but also induces respiration and acceleration of fruit ripening [30]. However, the fumigation with ethylene gas lacks flexibility and has high requirements for the tightness of storage cells and corresponding equipment. Besides, the application of ethephon is only permitted in the form of liberated ethylene gas [31]. Therefore, the ethylene microbubble (C_2_H_4_-MBs) technology has emerged recently, which allows accelerating the ripening of fruit in the form of dipping with the advantages of convenience and ease of operation [32].

#### 3.5.2. Strategy II—Reducing Ethylene for Climacteric Fruits

Strategy II mainly serves for climacteric fruits that need inhibition of ripening, the representative fruits include blueberry, apple, and peach. These fruits generally do not require further ripening treatment. To ensure good quality after long-term storage or long-distance transportation, it is necessary to reduce the microenvironment ethylene concentration, thereby suppressing the fruits’ physiological activities in general. The specific control methods include:

➣ Low temperature (such as cold chain transportation or cold storage) reduces the metabolic activities of fruits, thereby reducing ethylene biosynthesis and quality decay. This is currently the most widely used technical means, with the advantages of high efficiency and easy operation. However, the cold chain trucks or large cold storage may require a huge investment.
➣ Turning on the ethylene scrubbers could quickly and reliably reduce the ethylene concentration in the fruit’s microenvironment. Besides, the exhaust fans for ethylene scrubbers are convenient to integrate with the ethylene dynamic monitoring. This method is suitable for both storage and logistics processes.
➣ Spraying ethylene inhibitors. It is demonstrated that some ethylene inhibitors could antagonize with ethylene [33,34]. 1-Methylcyclopropene (1-MCP) is a popular ethylene inhibitor that prevents ethylene biosynthesis and furthering ethylene-dependent responses (including ripening and senescence of fruit tissues) by preventing ethylene to bind to the receptor [35,36,37,38,39]. Another ethylene inhibitor is the aminoethoxyvinylglycine (AVG), which inhibits the synthesis of ethylene by inhibiting the synthesis of ACC [40,41,42];
➣ Ethylene adsorbent, such as activated carbon, molecular sieve, potassium permanganate, and ethanol, could absorb the microenvironment ethylene gas. These adsorbents also easily integrate with fruit packages, thereby reducing ethylene contents in fruit’s independent packages.
➣ The microenvironment gas composition could be controlled with modified atmosphere packaging (MAP) for slowing down the quality decay and inhibiting the production of ethylene [43]. For example, low O_2_ and high CO_2_ in the microenvironment of fruit could inhibit ethylene biosynthesis and respiration, thereby delaying the ripening of fruit [2].

#### 3.5.3. Strategy III—For Non-Climacteric Fruits

Although ethylene was thought to play a limited role in the maturation and senescence of non-climacteric fruits before the 1970s, recent works have shown that ethylene has a close relationship with some quality evolution of non-climacteric fruits [44]. For non-climacteric fruits, ethylene does not induce its synthesis and response to exogenous ethylene treatment [45]. However, exogenous ethylene has a certain effect on some fruit color changes (such as de-greening for citrus fruits [46]) and might induce changes aroma compounds [36,47]. Since the impact of exogenous ethylene on non-climacteric fruits have not reached a consistent conclusion yet, it needs to be analyzed according to specific scenarios. For non-climacteric fruits that need to change color or enhance aroma, adding a small amount of ethylene may be useful, the specific control method usually includes the application of ethephon. Apart from that, the general recommendation for non-climacteric fruits is to limit the ethylene concentration.

## 4. Conclusions

The paper aimed to develop an ethylene sensor enabled dynamic monitoring and multi-strategies control for quality management of fruit cold chain. The solution could provide stakeholders with decision-making supports through remote, accurate, real-time collecting data of ethylene concentration in the fruit microenvironment and further improve the quality management level. The solution was verified with different fruit variety (blueberry, sweat cherry, and apple) and cold chain scenarios.

The results show that: (1) dynamic ethylene monitoring can monitor in real-time fruit microenvironment ethylene contents and upload data to a cloud terminal through the 4G network for users; (2) the response and recovery rate of electrochemical ethylene sensor can reach 2.3986 ppm/s and −2.4043 ppm/s, respectively, and 99% sensing accuracy could be achieved; (3) the quality parameters (firmness, pH, SSC, chromatic aberration, and rotting rate) evolution indicates the positive effect of multi-strategies ethylene control on improving fruit quality.

## Figures and Tables

**Table 1:** The set of characteristic parameters of the electrochemical ethylene sensor.

Type	No	Characteristic Parameters	Description	Value
Time-domain curve (Dynamic response curve)	1	X_a_ (ppm)	Baseline value in air	0
	2	X_g_ (ppm)	Stable response value	20.13
	3	X_m_ (ppm)	Maximum response value	20.40
	4	X_recair_ (ppm)	Final recovery value	0.03
	5	T_res_ (s)	Response time	18
	6	T_rec_ (s)	Recovery time	158
	7	T_Dres_ (s)	Duration from gas entry to D_res_	6
	8	T_Drec_ (s)	Duration from gas out to D_rec_	9
	9	T_Dresx_ (s)	Duration from gas entry to D_resx_	2
	10	T_Dresn_ (s)	Duration from gas entry to D_resn_	12
	11	T_Drecx_ (s)	Duration from gas out to D_recx_	3
	12	T_Drecn_ (s)	Duration from gas out to D_recn_	60
First-order derivative	12	D_res_ (ppm/s)	The maximum value of the first-order derivative in the response phase	2.3986
	13	D_rec_ (ppm/s)	The maximum value of the first-order derivative in the recovery phase	−2.4043
Second-order derivative	14	D_resx_ (ppm/s^2^)	Maximum value of the second-order derivative in the response phase	0.8586
	15	D_resn_ (ppm/s^2^)	The minimum value of the second-order derivative in the response phase	−0.0236
	16	D_recx_ (ppm/s^2^)	The maximum value of the second-order derivative in the recovery phase	−0.9979
	17	D_recn_ (ppm/s^2^)	The minimum value of the second-order derivative in the recovery phase	−0.0064
First-order integral	18	Int_T_ (ppm·s)	Signal integration in the period from gas entry to gas out	6963.72
	19	Int_Pres_ (ppm·s)	Signal integration in the period from gas entry to T_res_	475.96
	20	Int_Prec_ (ppm·s)	Signal integration in the period from gas entry to T_rec_	818.23

**Table 2:** Correlation analysis between the microenvironment ethylene concentration and the quality parameters of blueberry.

Gas content	Temperature	Firmness	SSC	pH	Rotting Rate
Ethylene	0 °C	−0.96	0.38	0.81	0.80
	5 °C	−0.98	0.12	0.57	0.86
	22 °C	−0.99	−0.51	−0.12	0.92

**Table 3:** Correlation analysis between the environment ethylene concentration and the quality parameters of sweet cherries.

Gas Content	Group	Firmness	SSC	pH	ΔE
Ethylene	I	−0.39274	−0.71885	0.691314	−0.33042
	II	−0.29544	−0.65403	0.770447	−0.22301
	III	−0.12128	−0.78234	0.90137	−0.18586

**Table 4:** Analysis of the ethylene multi-strategies control.

No	Control Strategy	Control Methods	Applicable Scenario	Typical Fruits	Applications	Main Advantages	Limitations
I	Adding ethylene	Fumigated with ethylene gas	Climacteric fruits that need to be ripened	Banana Mango Kiwi fruit	Storage	Automatically control. Direct action.	Complex devices, Costly
		Application of ethephon (ETH)			Storage/ Logistics	High Efficiency, Low-cost User-friendly.	Ethephon residual risk, Safety usage (strong acidity), Strict temperature control
		Dipped in ethylene microbubble			Storage	Convenient, Easy to carry out	Not Found Yet
II	Reducing ethylene	Low temperature	Climacteric fruits that need to inhibit ripen	Blueberry Peach Pear	Storage/ Logistics	High efficiency, User-convenient, Safe and eco-friendly.	Costly equipment
		Ventilation treatment			Storage/ Logistics	Easy operation, Automatic control	Limited effect
		Ethylene inhibitor (1-MCP or AVG)			Storage	High efficiency, Safe and eco-friendly, Prevention of chilling injury.	High concentration treatment may accelerate the deterioration of fruit quality
		Ethylene adsorbent			Storage/ Logistics	High efficiency, Less affected by temperature	Easy desorption, Poor adsorption capacity, Food safety risk
		Modified atmosphere packaging (MAP)			Logistics	Flexible packaging, Controllable gas composition	High cost
III	Adding ethylene	Application of ethephon (ETH)	Non-climacteric fruits	Cherries Strawberry Citrus	Storage/Logistics	Convenient User-friendly	Ethephon residual risk
